# Questionable Research Practices: A Principled Classification and Ranking Based on Survey Data

**DOI:** 10.1007/s11948-026-00589-w

**Published:** 2026-04-10

**Authors:** Daniele Fanelli, Alan Voodla, Siim Andres, Andero Uusberg

**Affiliations:** 1 Department of Research Methods and Practice, School of Social Sciences, Heriot-Watt University, Edinburgh, UK; 2 Department of Methodology, London School of Economics and Political Science, London, UK; 3 Institute of Psychology, University of Tartu, Tartu, Estonia; 4 Faculty of Psychology and Educational Sciences, KU Leuven, Belgium

**Keywords:** Questionable research practices, Misconduct, Taxonomy, Research integrity, Metascience

## Abstract

Questionable Research Practices (QRPs) are a highly diverse set of behaviours that eludes clear definition and demarcation. This study collects and organises definitions of QRPs given in surveys, and ranks them by frequency of reported engagement. We systematically retrieved surveys that asked researchers about their engagement with QRPs, and organised these survey definitions in two non-arbitrary classifications based, respectively, on the area of research affected by the QRP (e.g. authorship, data, analysis, etc…), and on the nature of the alteration of information entailed by the QRP (respectively, whether the QRP consisted in an omission, addition, or modification of information). Starting from this principled classification, we then created a list of non-overlapping QRP types that have been most commonly studied in surveys. We found that QRPs are more commonly reported when they pertain to the interpretation, analysis or citations, and when they involved the omission, rather than the addition or modification of information. Classified by type, the most commonly reported QRPs included “select analysis”, “select citation”, and “select covariates”; whilst the least commonly reported included “deny authorship”, and “FFP” (i.e. explicit fabrication, falsification, plagiarism, which are not QRPs but outright misconduct). Our QRP taxonomy and empirical results may find useful applications in research, training and policy-making.

**Supplementary Information:**

The online version contains supplementary material available at https://doi.org/10.1007/s11948-026-00589-w.

## Introduction

The notion of “Questionable Research Practice” (QRP) is central to the research integrity agenda and yet eludes clear definition and measurement. On the one hand, QRPs are more common than outright scientific misconduct (i.e. Fabrication, Falsification, and Plagiarism) and are believed to be potentially just as damaging if considerted in the aggregate. Therefore, they are seen as an important source of bias and irreproducibility that needs to be tackled with special training, incentives and punishments (e.g. Bouter, 2023). On the other hand, however, it is also well understood that QRPs are by definition a “gray area” of behaviours that may be valid of invalid depending on the context.

Indeed, the intrinsic difficulty in defining and demarcating QRPs generates considerable uncertainty and controversy about their actual prevalence and impact. In particular, most surveys might over-estimate the prevalence of QRPs by defining them too generically and by conflating the self-report of “engaging at least once” with the frequency of such behaviours in the literature (Fiedler & Schwarz, 2016). Furthermore, a growing theoretical and empirical literature suggests that under specific conditions, practices such as HARKing and selectively publishing results may have a neutral or even net positive impact on research progress (Rubin, 2017; de Winter & Happee, 2013). Similar arguments may be advanced for selectively reporting analyses, selectively citing the literature, dropping outliers and other QRPs that may be justified in some contexts (Fanelli, 2026), and that may be less harmful to research than currently believed (e.g. Ulrich & Miller, 2020).

### Challenges in Defining QRPs

A formal category of “Questionable Research Practices” was first proposed by a report elicited by the US government, as a way to separate outright misconduct from practices lacking a clear standard or consensus about their egregiousness (see Fanelli, 2011). This category was later dropped from the US federal definition of misconduct but is maintained in several contemporary research integrity guidelines and documents, albeit with different formal names - e.g. “unacceptable” practices (ALLEA, 2023). Notably, these documents do not offer an exact definition or classification of these practices, but rather they list examples of behaviours that, quoting the European Code of Conduct for Research Integrity : “In their most serious forms, […] are sanctionable, but at the very least every effort must be made to prevent, discourage, and stop them through training, supervision, and mentoring and through the development of a positive and supportive research environment” (ALLEA, 2023).

Formal classifications of QRPs have been proposed in the literature. Steneck (Steneck & Zinn, 2004), proposed to subdivide QRPs into nine core areas, based on the guidelines from the US Office of Research Integrity (ORI): “research misconduct”, “protection of human subjects, “welfare of laboratory animals”, “conflicts of interest, “data management practices”, “mentor and trainee responsibilities”, “collaborative research”, “authorship and publication”, and “peer review”. This classification is comprehensive, as it covers several aspects of research procedures, but it is limited in scope since it is not a principled taxonomy (i.e. it is not based on a scheme or classification hierarchy), it is primarily relevant to biomedical research (which is the focus of ORI), and it is rather coarse-grained with regards to behaviours that may distort scientific knowledge, which are a core concern of the research integrity agenda (Bouter, 2023).

A more principled “taxonomy of research misconduct” was offered by Hall and Martin (2019), who qualified four classes of research behaviour (“data manipulation”, “use of work by others”, “user of own work”, and “authorship”) along four levels of severity: “appropriate conduct”, “questionable conduct”, “inappropriate conduct” and “blatant misconduct”. This scheme has the merit of being based on two clearly defined dimensions, but its severity criteria follow a somewhat arbitrary standard: the level of gravity of a practice is defined by whether or not “clear rules” are being infringed. In particular, it defines “inappropriate” conducts as infringing rules that are subject to “some variation by field, country, institution and/or journal”, whereas it defines “questionable” conducts as having no clear rules against them but failing the test of a “reasonable reader”, such that their perpetrator would be “embarrassed/reluctant to be revealed”. In most research fields, rules are either non-existent or defined tacitly, and therefore it is hard to apply Hall and Martin’s taxonomy consistently across research contexts.

A different taxonomy was offered by Manapat et al., (2024), whose main objective was to clarify the distinction between QRPs and “Researchers degrees of freedom” (RDF, which refers to the flexibility of methodological practices). They consider primarily two binary dimensions: whether “the action taken was defensible” rather than not, and whether “all the necessary/relevant information has been acquired” rather than not, and they superimpose this on a framework that distinguishes between “research practices” and “reporting practices”. The result splits research practices into three levels (Single Best Option, RDF, and QRP), and reporting practices into five levels (three types of “honest practices”, in which reporting may or may not be complete and correct, and two types of “fraudulent” practices, i.e. Fabrication and Falsification). This scheme is based on relatively well-defined criteria, but it yields counter-intuitive results. For example, according to this scheme, we should classify as a QRP not only a research action that is not defensible, but also an action that is defensible but is reported incompletely – in other words, it potentially classifies as QRPs practices that are scientifically sound and that do not distort the scientific record, an approach that might obfuscate, rather than clarify, the demarcation criteria for QRPs. Moreover, this scheme appears to be of limited use outside the literature for which it was conceived, as it is too coarse-grained to capture the diversity of practices that are typically of interest to research, education, or policy-making on QRPs.

### Challenges in Measuring QRP Frequency

The difficulty of devising clear and non-arbitrary definitions of QRPs translates into even greater difficulties in estimating the true prevalence and impact of such problematic behaviours. This is not for lack of data, which has been produced in abundance, particularly in the form of surveys asking researchers about their engagement with questionable practices. However, due to the lack of a univocal classification, surveys will typically list a variety of different behaviours, only a few of which are common across more than a few surveys, and will use their own phrasings and standards to define each QRP. Combined with other sources of heterogeneity, this impedes the meta-analytical pooling of survey data to produce generalisable estimates (Fanelli, 2009). A recent meta-analysis tried to pool survey data on QRPs (Xie et al., 2021), but it did so using the categories proposed by Steneck & Zinn, (2004), which, as discussed above, are insufficiently fine-grained to enable meaningful comparisons between different forms of questionable practice.

### Current Study

This study pools survey data using a novel, data-driven but also principled classification scheme. Specifically, we had three main objectives: 1) Document the practices that surveys on QRPs have attempted to measure; 2) Organize the definitions of QRPs given in these surveys into a set of mutually exclusive and internally consistent categories; 3) Summarize data on the prevalence of each resulting category of QRP, pooling data across surveys.

Our central aim was to produce an adequately fine-grained and theoretically meaningful classification of QRPs that overcomes the limitations of previously proposed taxonomies. To this end, we centred our classification approach around the concept of information, which has been applied to other meta-scientific problems with encouraging results (e.g. Fanelli, 2019; Fanelli et al., 2025). In particular, we posited that a research practice is questionable to the extent that it entails either the omission, the addition, or the manipulation of information, where information is intended in the broadest possible sense of any “difference that makes a difference” (Bateson, 1972) - that is, any element presented in a study that makes a difference to what the study communicates to a given field of research, either directly (e.g. data, methods, interpretation) or indirectly (e.g. authorship, acknowledgments, Conflicts Of Interest). We overlap this classification logic with one based on the area of research that the information manipulation applies to (e.g. data, analysis, results, authorship etc.). Finally, partially based on this two-dimensional classification, we proceeded to also classify QRPs into a set of mutually exclusive and descriptive categories, which allows us to assess what types of QRPs are most commonly reported in surveys.

## Methods

### Literature Search and Selection

Our objective was to collect explicit definitions of “questionable research practices” used in surveys and the corresponding frequency with which respondents reported engaging with them. We thus included studies that satisfied the following criteria:

- The study is about practices explicitly defined by the phrase “questionable research practices” or the acronym QRP.
- The study reports original data (i.e. is not a review or a meta-analysis).
- The data were collected via a survey (i.e. a questionnaire administered to respondents).
- The survey included questions that asked how often the respondents had engaged in specific behaviours.

On the 8th of March 2023, we searched the Web of Science Core Collection database with the boolean string “(‘Questionable Research Practices’ OR ‘QRP’) AND ‘survey’ “, within title, abstract, or keywords. Results were limited to the following record categories: article, review, and editorial material.

This searched yielded a preliminary list of *n* = 72 unique records. Inspection of their abstracts identified 48 potentially relevant studies of which the PDF was retrieved and inspected, leading to a final list of 20 studies that were included in the analysis.

### Data Collection

From each potentially relevant question about QRPs in each of the included studies, we recorded the text of each question *verbatim*, the formulation of the question itself, the percentage of respondents who reported engaging in the corresponding QRP at least once, and other study and question characteristics.

#### Outcome Extraction

Included surveys used a variety of outcome scales to measure the frequency at which respondents engaged in QRPs. In order to make results comparable across surveys with different outcome scales, for each QRP in each study we recorded the percentage of respondents who reported engaging in it at least once - this is the quantity that we refer to as “admission rate”. When this number was not provided directly in the survey, it was calculated following the methods used in Fanelli (2009) – that is, by subtracting from 100 the sum of the percentages of respondents who either did not respond to the question (e.g. answering “don’t know”) or who answered that they had never engaged in it (e.g. “never” “zero” etc.).

In all but one of the included studies, the necessary percentage data was provided directly in numerical form. The one exception (Fiedler & Schwarz, 2016) presented results exclusively in a bar chart, and data from this study was extracted using a freely available plot digitizing software (https://apps.automeris.io/wpd/).

When studies presented data both in aggregated form and broken down into subsets (for example, presenting first the global percentage and then the percentage of respondents at different career levels) we recorded the data at the subsample level, in order to obtain the most fine-grained information.

#### Study and Question Characteristics

Additional data recorded for each question and study included:

1. Sample size: the total number of survey respondents, which gives the baseline to calculate the admission rate.
2. The target sample size: the number of participants that were invited to the survey (e.g. the total number of emails sent out, minus the rejected emails), which allowed us to calculate the response rate for most studies.
3. Discipline of respondents: the research area of the respondents targeted for the survey, if specified.
4. Country of respondents: the country or countries in which the survey was conducted, if specified.
5. Sample demographics: any relevant characteristic reported about the study sample. Typically, studies specified the participants’ career levels, and often would provide other descriptive characteristics of the sample (e.g. gender, mean age, etc.). We recorded any information available, in order to assess later what variables could be used for between-study comparisons.
6. Sampling methodology: as we did for sample demographics, we recorded any relevant information concerning how the sample of participants had been determined – for example, whether the survey targeted participants from a particular institution, or country, and/or whether it was a random sample or not, etc.
7. Survey method: in particular, we recorded how participants were invited to the survey and how the survey was administered since survey administration method was shown in a previous meta-analysis to be associated with different admission rates (Fanelli, 2009).
8. Outcome scale: the scale used to measure the frequency of engagement with the QRP, from which the percentage of non-engagement had been extracted (as described above).

We subsequently recoded the data collected for characteristics 3–7 as follows:

- Discipline: discipline was categorized using the Essential Science Indicators system, which includes 21 mutually exclusive disciplinary categories and had been used in multiple previous meta-scientific studies (e.g. Fanelli, 2010; Fanelli et al., 2017).
- Domain: whether the discipline pertained to the Physical, Biological, or Social sciences, obtained by aggregating the ESI disciplines.
- Country: samples either came from one of eight countries (Australia, Canada, Croatia, Germany, Italy, Netherlands, Norway, USA) or were multinational.
- Continent: based on the list of countries specified for each sample (where available), data was aggregated into the following categories: America, Europe, Oceania, and Mixed. Since Oceania only had 8 studies and insufficient overlap of QRP categories with the rest of the sample, it was merged with “mixed” in a “mixed/other” category.
- Career level: based on the descriptions given, samples or subsamples of participants could be classified as one of the following: Undergraduate (UG) student, Masters (MSc) student, PhD student, researcher (i.e. any active researcher, assistant, associate, or full professor, with a PhD), or mixed. Samples in which students or graduate students constituted a net minority (e.g. less than 10%) were classified as “researcher”. However, since few studies included non-PhD students, this category was subsequently recoded as a ternary variable separating “pre-PhD” respondents (including graduate students), “post-PhD” respondents, and “mixed” samples.
- Outcome scale range: based on the description of the options given to respondents, we classified the scale in terms of the number of options available, which varied between 2 (binary choice) and 7.
- Recall period: based on the question asked, we recorded a binary variable reflecting whether the recall period was unlimited (e.g. questions asking “Did you ever engage in …”) or whether it was limited. Surveys that specified a limited recall period had all used three years as the threshold.

The screening of PDFs and the data extraction were performed by SA and AV, and the resulting data were checked and corrected where necessary, and then recoded and classified by DF with feedback from all other co-authors.

### Classification of QRPs

We classified QRPs along three dimensions, where the first two are principled dimensions that together form a taxonomy, and the third is a descriptive classification that separates the specific types of QRPs that surveys have investigated.

1) Area: separates QRPs by the area of research they pertained to. After a few iterations, we identified the following areas:

- Methods
- Data
- Analysis
- Results
- Interpretation
- Limitations
- Publication
- Credit (including authorship)
- Peer-review
- Citations
- Relations (that is, professional and collaboration relationships)
- Conflicts Of Interest (COI)
- Ethics (breaches pertaining to research ethics requirements other than COI)
- FFP (questions that asked specifically about data fabrication, falsification, and plagiarism, which are not QRPs).

2) Modality: separates behaviours according to three simple and objective qualifiers reflecting the nature of the misrepresentation of information:

- Omission: the QRP definition entails concealing relevant information (e.g. dropping data point or covariates from an analysis, not citing contrary evidence, denying credit to a deserving author).
- Addition: the QRP definition entails adding information (e.g. collecting new data after checking results, running a new analysis post-hoc, or gifting authorship).
- Modification: the QRP definition entails active modification of information (e.g. improperly rounding-down a *p*-value, misreporting methods, deliberately citing work incorrectly).
- Other: A few QRPs that were not easily classifiable as any of the above categories (e.g. misappropriating research resources).

3) Type: partially based on the cross-classification of area and modality, merging a few such categories and renaming others, this third classification contains relatively homogeneous sets of behaviours that recurred in surveys. This classification descriptively captures the variability of practices actually examined in surveys. The full list of types is given in Fig. 2 in the results section.

The full set of QRP descriptions extracted from surveys, with the exact original wording and their subsequent classification is available in the Supplementary Information.

### Analyses

Due to the highly heterogeneous nature of the data sources, our main analyses are non-parametric. The statistical significance of median differences between categories is assessed with a median test and linear relations between continuous variables with bivariate regression.

The rank ordering of QRPs is the inverse order of the median percentage admission rate (the value that divides the data into two equally sized parts). For this rank order, we also calculated the non-weighted average percentage rate for each category and a weighted mean according to meta-analytical standards (by first transforming the percentages in logits, calculating the corresponding inverse-variance weight, then pooling the result and back-transforming it to the mean percentage).

When comparing QRP frequencies between categories of participants, we restricted the sample to QRP categories that had at least two data points in each category.

Three survey studies yielded more than one subset of data (e.g. same questions asked to different categories of participants), which makes data from those subsets not statistically independent. Therefore, statistical tests were performed after retaining a single subset for each study, yielding more conservative statistical estimates.

### Inter-Coder Reliability

All QRP phrases were initially classified by DF. Then the inter-coder reliability (IRR) of the three classification methods (i.e. by area, modality and type) were assessed among the three remaining authors (AU, AV, SA) using Krippendorff’s alpha (Krippendorff, 2019) implemented by the r package “icr” (Staudt et al., 2024). A conventional threshold of reliability for this measure is considered to be 0.8, with values equal or above 0.67 considered acceptable for tentative results. A first random sample of *N* = 24 QRPs was classified with no guidance, reaching an IRR of 0.5, 0.37, and 0.6, respectively. After corrections and explanations by DF, the second round (*N* = 23) obtained IRRs of 0.58, 0. 65 and 0.81, respectively. Pairwise calculations showed that IRRs of 0.65 or more were obtained by one pair of coders in all cases, with a maximum IRR of 0.91 for the third category.

The IRR estimates suggested that basic instructions and a modest amount of practice allows any two researchers to reach high reliability. Rather than continue the coder training to further increase IRR, we proceeded to have all remaining QRPs double-coded by a second rater (i.e. AU, AV and SA, each coded one third of remaining data) and any inconsistencies with DF’s initial classification were examined and moderated.

The moderation led to revision of some of the categories. First we removed “FFP” as a separate QRP area and distributed instances of FFP to the remaining QRP areas, mainly to the areas of “credit” and “data”. The FFP category is retained in the QRP type classification. Second, we merged “other” and “other, ethics” QRP types into a single “other” type, as this distinction was deemed unnecessary.

All results are calculated on these final, moderated and adapted classifications. The entire process, including the independent coding of all data and the final moderated classification, is documented in the data file shared as Supplementary Information.

## Results

The final sample size comprised 296 QRP data points, obtained from 23 separate samples, collected in 20 published studies. Study and sample characteristics are shown in Table 1.

**Table 1:** Characteristics of included studies: Author first name, year of publication, country in which the survey was conducted, discipline of respondents, career level of respondents, number of responses, and literature reference

First aut.	Year	Country	Discpline	Career level	N	Ref.
John	2012	USA	psychology	mixed	2155	John et al., 2012
Braun	2012	mixed	psychology	researcher	257	Braun & Roussos, (2012)
Rajah-Kanagasabai	2015	Australia	psychology	UG student	205	Rajah-Kanagasabai & Roberts, (2015)
Fiedler	2016	Germany	psychology	researcher	1138	Fiedler & Schwarz, (2016)
Agnoli	2017	Italy	psychology	researcher	208	Agnoli et al., (2017)
Pupovac	2017	Croatia	mixed	researcher	237	Pupovac et al., (2017)
Motyl	2017	mixed	psychology	mixed	1166	Motyl et al., (2017)
Felaefel	2018	mixed	medicine	mixed	224	Felaefel et al., (2017)
Fraser	2018	mixed	ecology	researcher	807	Fraser et al., (2018)
Artino	2019	mixed	medicine	mixed	589	Artino et al., (2019)
Makel	2021	mixed	education	researcher	1488	Makel et al., (2021)
Bakker	2021	mixed	communication	researcher	872	Bakker et al., (2021)
Ljubenkovic	2021	Croatia	medicine	UG student	220	Ljubenković et al., (2021)
Ferraro	2022	mixed	economics	mixed	393	Ferraro & Shukla, (2022)
Isbell	2022	USA	linguistics	mixed	322	Isbell et al., (2022)
Gopalakrishna	2022	Netherlands	mixed	mixed	6813	Gopalakrishna et al., (2022)
Swift	2022	USA	psychology	researcher	164	Swift et al., (2022)
Swift	2022	USA	psychology	PhD student	110	Swift et al., (2022)
Kaiser	2022	Norway	mixed	researcher	7291	Kaiser et al., (2021)
Moran	2023	Canada	psychology	PhD student	168	Moran et al., (2023)
Moran	2023	Canada	psychology	MSc student	81	Moran et al., (2023)
Moran	2023	Canada	psychology	UG student	171	Moran et al., (2023)
Chin	2023	mixed	criminology	researcher	1612	Chin et al., (2021)

**Fig. 1:**
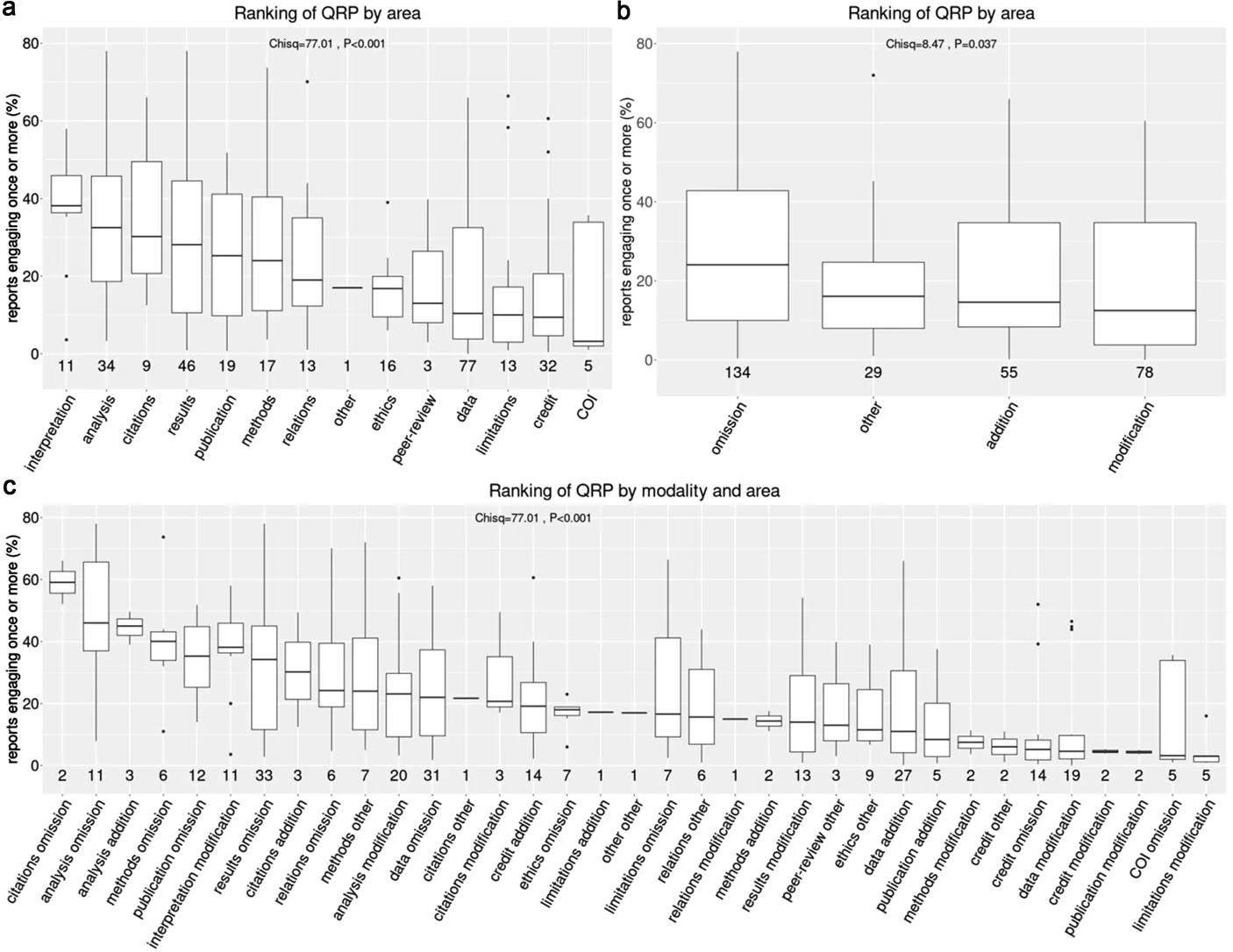
Boxplots of the percentage of engagement (once or more) with QRP, classified by relevant area of research and by modality (classified in terms of whether there was omission, addition or modification of information – NA are non-classifiable behaviours). Boxplots show median, interquartile range and outliers

Classified by QRP area and ranked by median reporting rate, the most commonly reported QRPs pertain to the areas of interpretation, analysis, citation, and results, whereas the least commonly reported QRPs pertain to limitations, credit (that is, authorship and acknowledgement) and conflicts of interests (Fig. 1a).

Classified by QRP modality, the most commonly reported QRPs involved omission of information (e.g. dropping data, omitting citations), whilst the other modalities were reported at similar frequencies (Fig. 1b).

 Intersecting area and modality gives rise to a fine-grained principle classification, that unequivocally indicates the omission of citations as the most common QRP, followed by omissions or additions in the analysis, whereas modifying publications, omitting conflicts of interest information, and modifying information concerning the limitations of a study are the least commonly reported (Fig. 1c).

**Fig. 2:**
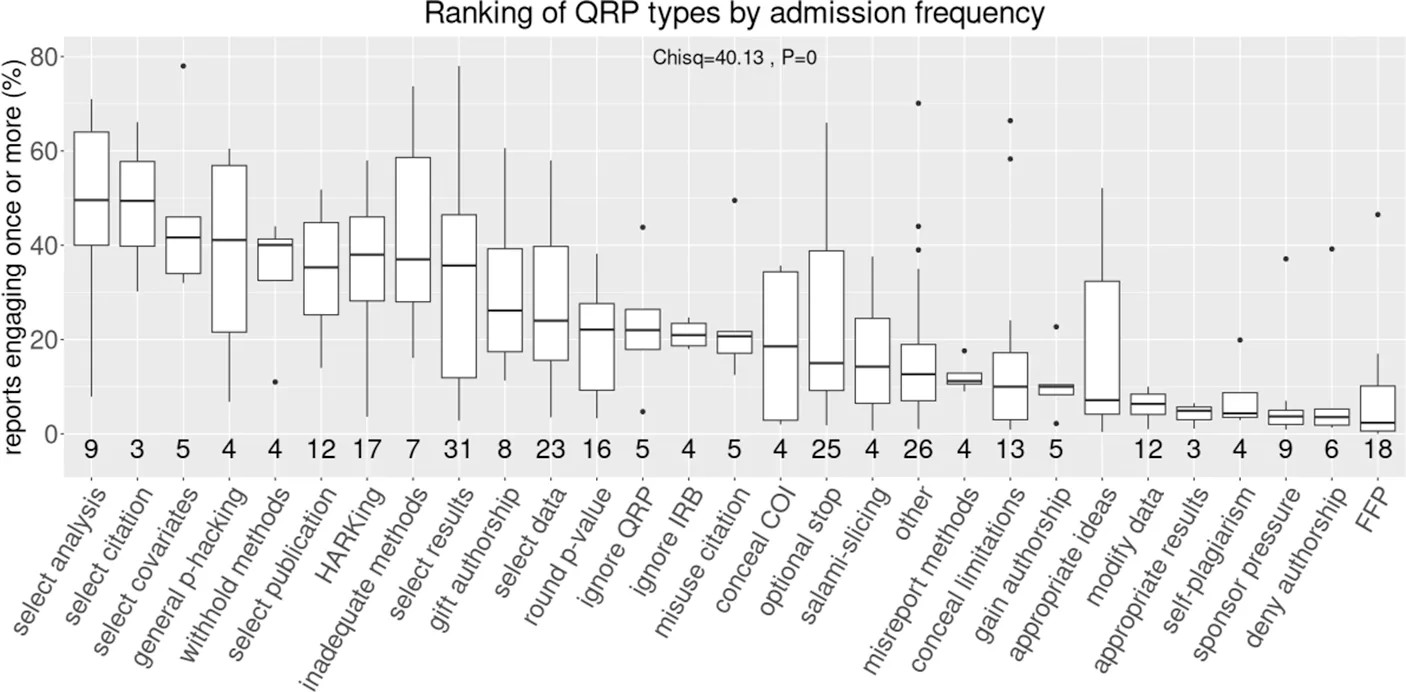
Median and weighted mean reported percentages of QRPs. Boxplots show median, interquartile range and outliers. QRPs are coloured by area and ordered by decreasing median value. Red triangle show the corresponding weighted-mean value. Numbers above the labels correspond to the number of surveys (data points) for each category

Finally, our classification by QRP type identified 27 QRP categories, plus an FFP category for outright misconduct (i.e. descriptions of QRPs that openly use the terms “fabrication”, “falsification”, and “plagiarism”, and are therefore technically not QRPs), and an “other” category to collect the remaining, heterogeneous behaviours. Ranked by inverse median, the most commonly reported QRP types include selecting the analysis or the covariates within the analysis, selectively citing sources, withholding methodological details and HARKing (acronym for Hypothesizing After Results are Known). At the other extreme of the spectrum, we find FFP (i.e. behaviours that qualify explicitly as scientific misconduct), denying authorship to someone who deserved it, and changing one’s methods or results in response to a funding source (Fig. 2).

### Robustness and Secondary Analyses

The overall median admission rate did not vary significantly between surveys using different distribution methods or administration methods (Fig. S1, top panels). Overall admission rates were also not linearly associated with sample size and showed a negative relation with response rates, which however was not significant controlling for sample size. However, surveys that specified a recall period of three years had higher median admission than surveys not specifying a recall period (Fig. S1, centre left), a trend that was observable even within most QRP categories. Survey results also differed across different outcome scale ranges but with no clear pattern (Fig. S1, centre right).

These differences may reflect genuine methodological effects, but may also arise at least in part from important confounding effects, caused by the heterogeneity of included surveys.

A further indication of survey heterogeneity was observable when subdividing surveys by participant characteristics (Fig. S2). In particular, some QRPs were admitted more commonly in surveys of undergraduate, graduate, and doctoral students compared to post-PhD researchers (Figure S2, top). Higher admission rates were also observed in the biomedical sciences relative to the social sciences or multidisciplinary samples (Figure S2, center), and in participants in Europe relative to the USA (Figure S2, bottom).

Since these categories overlap with each other and with other methodological characteristics of surveys, they cannot be generalized or taken as direct evidence of differences in the rate of QRPs. However, they suggest that the rank ordering in Fig. 2 may be confounded by multiple sources of survey heterogeneity.

Differences in reporting rates between career levels are an especially important potential confounding factor since data amongst undergraduate and graduate students shows unusually high levels of reported FFP, which is at odds with all previous evidence on the frequency of such behaviours (Fanelli, 2009; Pupovac & Fanelli, 2015). To assess the impact of this latter potential confounding factor, we recalculated the ranking separately for surveys conducted amongst pre-PhD respondents, post-PhD respondents, and samples mixing all career levels. Results in the latter two subsets are similar to each other (Figure S3 centre and bottom) and suggest that “selecting analysis”, “Harking”, “selecting results”, “selecting covariates”, “selecting publications”, “poor methods” and “gift authorship” are the top-most commonly reported QRPs.

## Discussion

To the best of our knowledge, this is the first study to systematically document, organize, and summarise definitions and data about QRPs investigated in surveys. It uses a novel and principled approach, which posits that a QRP consists in either the omission, addition, or modification of information pertaining to the various components of research (which we call “area”, Fig. 1). By superimposing these two non-arbitrary taxonomical dimensions (i.e. the modality of information distortion and the relevant research QRP area) a suitably fine-grained and theoretically meaningful classification emerges (Fig. 1c). This classification in principle comprises 56 categories (i.e. 14 areas, each with 4 modalities), but in practice many theoretical categories are not covered by the empirical QRP surveys we analysed, and many categories are represented by very few data points.

In addition to our principled classification, we produced a descriptive classification of the QRP types most commonly reported in surveys. This comprises 27 distinct and mutually exclusive QRP types, which reflect the practices that surveys on QRPs have been concerned with (Fig. 2).

Our empirical results revealed several patterns in QRP admission rates, but these must be taken with great caution, due to the significant limitations of the data available. The first limitation concerns the high heterogeneity of samples and survey methods, which is a known and important limitation of survey data on scientific integrity (Fanelli, 2009; Xie et al., 2021). Therefore, whilst the differences observed between survey subsets (i.e. Fig. S1, S2) could reflect genuine behavioural differences between different types of participants, the number of possible variables and confounding effects is simply too large to control, limiting conclusions.

A second limitation of this analysis concerns the metric used to compare survey results. Similarly to previous quantitative reviews (Fanelli, 2009; Pupovac & Fanelli, 2015; Xie et al., 2021), we standardized results across surveys by calculating the percentage of respondents who reported having engaged in a QRP “at least once”. This is the only viable approach to pool these data, but we agree with previous critiques (e.g. Fiedler & Schwarz 2016) suggesting that this method yields a very imperfect measure of QRP prevalence and it gives no information about the relative impact of QRPs on the scientific literature.

A third limitation of the data pertains to study choices that may lead to data inaccuracies. In particular, we noticed that surveys conducted amongst undergraduate students showed incongruous results and implausibly high levels of FFP, suggesting that the most reliable results are obtained by excluding undergraduate respondents.

Finally, our retrieval of surveys was limited to one database. Whilst we believe that this search has captured the vast majority of surveys on QRPs, it is nonetheless possible that a few such surveys were missed. Other surveys on QRP have been published since we conducted the search for this study, and future studies might consider extending our analysis to other survey data.

Despite these limitations, our analysis detected a rank-ordering of QRP by frequency that was robust to inclusion criteria. Based on our analysis, we suggest that this rank ordering is largely explained by three factors:

1) Omission vs commission: the most frequently reported QRPs tend to be the ones that entail omission, rather than modification or addition of information, across QRP areas (Fig. 1b). This result aligns with experiments suggesting that lying by omission is generally perceived to be less ethically problematic than lying by commission (van Swol et al., 2022). It also validates the assumptions of past theoretical models that equated QRP with lying by omission and misconduct with commission (Gall & Maniadis, 2019).

2) Ethical ambiguity: many of the highly reported QRPs tend to be the ones whose problematic nature is most ambiguous and context-dependent, including HARKing, p-hacking and selectively presenting analyses or results (see Rubin 2017; Mohseni, 2020; Ulrich & Miller 2020; Fanelli 2026).

3) Boundary arbitrariness: some of the highly reported QRPs have arbitrary or subjective boundaries. For example, the dividing line between legitimate authorship and Gift Authorship (i.e. determining whether a colleague has made a sufficient contribution to the study to deserve authorship) is in many cases subjective and influenced by field and cultural conventions.

These three factors characterise practices that are either perceived to be less harmful or indeed that represent justified methodological choices. The most commonly reported practices characterised by these factors may thus not be, as often suggested, a priority concern for research integrity, at least in terms of their potential to distort the literature. Therefore, a Research Integrity agenda that aims to prioritize the prevention and correction of QRPs that are most damaging to research may not meet its objectives if it focuses on the QRPs that are most commonly reported.

Besides our empirical results - which, as we emphasise, have important limitations - the key contribution of our study is a novel classification approach to QRPs. In particular, our system based on two purely descriptive dimensions (the area and the modality of a QRP) is theoretically derived and more fine-grained than current alternatives (Hall & Martin, 2019; Manapat et al., 2024). Moreover, this classification may easily and systematically be applied to other questionable research practices, especially if these can be defined in terms of how they entail omission, addition of modification of information. In some cases, this may invite re-defining QRPs in more specific terms. For example, generic definitions like “inadequate mentoring of junior colleagues” may be accommodated in this classification by specifying one or more ways in which information is inadequately transmitted by the mentor to the mentee. Re-defining research practices this way may be a valuable exercise that clarifies what is meant by otherwise generic terms like “inadequate” or “poor” that are often used to define QRPs. As it increases the clarity and specificity of QRP definitions, we believe that this multidimensional classification of QRPs may have useful applications in education, policy-making, and research on research integrity.

## Electronic supplementary material

Below is the link to the electronic supplementary material.

Supplementary Material 1

Supplementary Material 2

Supplementary Material 3

## Data Availability

The raw data and R code script underlying these results are included as electronic supplement to the article.

## References

[CR1] Agnoli, F., Wicherts, J. M., Veldkamp, C. L., Albiero, P., & Cubelli, R. (2017). Questionable research practices among Italian research psychologists. *PLoS ONE, * 12. 10.1371/journal.pone.0172792 PMC5351839 28296929

[CR2] ALLEA, A. E. A. (2023). The European Code of conduct for research Integrity.

[CR3] Artino, A. R., Driessen, E. W., & Maggio, L. A. (2019). Ethical shades of gray: International frequency of scientific misconduct and questionable research practices in health professions education. *Academic Medicine*, *94*, 76–84. 10.1097/ACM.0000000000002412 30113363

[CR4] Bakker, B. N., Jaidka, K., Dörr, T., Fasching, N., & Lelkes, Y. (2021). Questionable and open research practices: Attitudes and perceptions among quantitative communication researchers. *Journal of Communication*, *71*, 715–738.

[CR44] Bateson, G. (1972). Steps to an ecology of mind: *Collected essays in anthropology, psychiatry, evolution, and epistemology*. Jason Aronson Inc.

[CR6] Bouter, L. (2023). Research misconduct and questionable research practices form a continuum. *Accountability in Research*, 1–5. 10.1080/08989621.2023.2185141 36866641

[CR7] Braun, M., & Roussos, A. J. (2012). Psychotherapy researchers: Reported misbehaviors and opinions. *Journal of Empirical Research on Human Research Ethics*, *7*, 25–29. 10.1525/jer.2012.7.5.25 23324200

[CR8] Chin, J. M., Pickett, J. T., Vazire, S., & Holcombe, A. O. (2021). Questionable research practices and open science in quantitative criminology. *Journal of Quantitative Criminology*, *39*, 21–51.

[CR9] de Winter, J., & Happee, R. (2013). Why selective publication of statistically significant results can be effective. *PLoS ONE*, *8*, e66463. 10.1371/journal.pone.0066463 PMC3688764 23840479

[CR10] Fanelli, D. (2009). How many scientists fabricate and falsify research? A systematic review and meta-analysis of survey data. *PLoS ONE*, *4*, e 5738. 10.1371/journal.pone.0005738 PMC2685008 19478950

[CR11] Fanelli, D. (2010). ‘Positive’ results increase down the hierarchy of the sciences. *PLoS ONE*, *5*, e10068. 10.1371/journal.pone.0010068 PMC2850928 20383332

[CR45] Fanelli, D. (2011). The black, the white and the grey areas: Towards an international and interdisciplinary definition of scientific misconduct. In T. Meyer & N. H. Steneck (Eds.), *Promoting research integrity on a global basis*. World Scientific.

[CR13] Fanelli, D. (2019). *A theory and methodology to quantify knowledge* (p. 6). Royal Society Open Science. 10.1098/rsos.181055 PMC6502358 31183113

[CR14] Fanelli, D. (2026). Towards a theoretical framework for metascience: When doquestionable research practices hurt the most? (under review).

[CR15] Fanelli, D., Costas, R., & Ioannidis, J. P. A. (2017). Meta-assessment of bias in science. In *Proceedings of the National Academy of Sciences* (Vol. 114, pp. 3714–3719). 10.1073/pnas.1618569114 PMC5389310 28320937

[CR16] Fanelli, D., Tan, P. B., Amaral, O. B., & Neves, K. (2025). *A metric of knowledge as information compression reflects reproducibility predictions for biomedical experiments* (p. 12). Royal Society Open Science. 10.1098/rsos.241446 PMC12312647 40746963

[CR17] Felaefel, M., Salem, M., Jaafar, R., Jassim, G., Edwards, H., Rashid-Doubell, F., Yousri, R., Ali, N. M., & Silverman, H. (2017). A Cross-sectional survey study to assess prevalence and attitudes regarding research misconduct among investigators in the Middle East. *Journal of Academic Ethics*. 10.1007/s10805-017-9295-9 PMC5945220 29755305

[CR18] Ferraro, P. J., & Shukla, P. (2022). Credibility crisis in agricultural economics. *Applied Economic Perspectives and Policy*, *45*, 1275–1291.

[CR19] Fiedler, K., & Schwarz, N. (2016). Questionable research practices revisited.* Social Psychological and Personality Science*, *7*, 45–52.

[CR20] Fraser, H., Parker, T., Nakagawa, S., Barnett, A., & Fidler, F. (2018). Questionable research practices in ecology and evolution. *PLoS ONE*, *13*, e0200303. 10.1371/journal.pone.0200303 PMC6047784 30011289

[CR21] Gall, T., & Maniadis, Z. (2019). Evaluating solutions to the problem of false positives. *Research Policy*, *48*, 506–515.

[CR22] Gopalakrishna, G., Riet, G., Vink, G., Stoop, I., Wicherts, J. M., & Bouter, L. M. (2022). Prevalence of questionable research practices, research misconduct and their potential explanatory factors: A survey among academic researchers in the Netherlands. *PLoS ONE*, *17*, e0263023. 10.1371/journal.pone.0263023 PMC8849616 35171921

[CR23] Hall, J., & Martin, B. R. (2019). Towards a taxonomy of research misconduct: The case of business school research. *Research Policy*, *48*, 414–427.

[CR24] Isbell, D. R., Brown, D., Chen, M., Derrick, D. J., Ghanem, R., Arvizu, M. N. G., Schnur, E., Zhang, M., & Plonsky, L. (2022). Misconduct and questionable research practices: The ethics of quantitative data handling and reporting in applied linguistics. *Modern Language Journal*, *106*, 172–195.

[CR43] John, L. K., Loewenstein, G., & Prelec, D. (2012). Measuring the prevalence of questionable research practices with incentives for truth telling. *Psychological Science*, *23*(5), 524–532. 10.1177/0956797611430953 22508865

[CR25] Kaiser, M., Drivdal, L., Hjellbrekke, J., Ingierd, H., & Rekdal, O. B. (2021). Questionable research practices and misconduct among Norwegian researchers. *Science and Engineering Ethics*, 28 10.1007/s11948-021-00351-4 PMC8692305 34932191

[CR26] Krippendorff, K. (2019). *Content analysis*: *An**introduction to its methodology*. SAGE Publications, Inc.

[CR27] Ljubenković, A. M., Borovečki, A., Ćurković, M., Hofmann, B., & Holm, S. (2021). Survey on the research misconduct and questionable research practices of medical students, PhD students, and supervisors at the Zagreb School of Medicine in Croatia. *Journal of Empirical Research on Human Research Ethics*, *16*, 435–449. 10.1177/15562646211033727 34310249

[CR28] Makel, M. C., Hodges, J., Cook, B. G., & Plucker, J. A. (2021). Both questionable and open research practices are prevalent in education research. *Educational Researcher*, *50*, 493–504.

[CR29] Manapat, P. D., Anderson, S. F., & Edwards, M. C. (2024). A revised and expanded taxonomy for understanding heterogeneity in research and reporting practices. *Psychological Methods*, *29*, 350–361. 10.1037/met0000488 35404630

[CR30] Mohseni, A. (2020). Harking: From misdiagnosis to misprescription. *PhilSci Archive*, https://philsci-archive.pitt.edu/id/eprint/18523. https://philsci-archive.pitt.edu/id/eprint/18523

[CR31] Moran, C., Richard, A., Wilson, K., Twomey, R., & Coroiu, A. (2023). I know it’s bad, but I have been pressured into it: Questionable research practices among psychology students in Canada. *Canadian Psychology/Psychologie Canadienne*, *64*, 12–24.

[CR32] Motyl, M., Demos, A. P., Carsel, T. S., Hanson, B. E., Melton, Z. J., Mueller, A. B., Prims, J. P., Sun, J., Washburn, A. N., Wong, K. M., Yantis, C., & Skitka, L. J. (2017). The state of social and personality science: Rotten to the core, not so bad, getting better, or getting worse? *Journal of Personality & Social Psychology*, *113*, 34–58. 10.1037/pspa0000084 28447837

[CR33] Pupovac, V., & Fanelli, D. (2015). Scientists admitting to plagiarism: A meta-analysis of surveys. *Science and Engineering Ethics*, *21*, 1331–1352. 10.1007/s11948-014-9600-6 25352123

[CR34] Pupovac, V., Prijić-Samaržija, S., & Petrovečki, M. (2017). Research misconduct in the Croatian scientific community: A survey assessing the forms and characteristics of research misconduct. *Science and Engineering Ethics*, *23*, 165–181. 10.1007/s11948-016-9767-0 26940319

[CR35] Rajah-Kanagasabai, C. J., & Roberts, L. D. (2015). Predicting self-reported research misconduct and questionable research practices in university students using an augmented theory of planned behavior. *Frontiers in Psychology,* 6. 10.3389/fpsyg.2015.00535 PMC4415326 25983709

[CR36] Rubin, M. (2017). When does HARKing hurt? Identifying When different types of undisclosed post hoc hypothesizing harm scientific progress. *Review of General Psychology*, *21*, 308–320.

[CR37] Staudt, A., L’Ecuyer, P., & Chan, C.-H. (2024). icr - Compute Krippendorff’s Alpha.

[CR38] Steneck, N., & Zinn, D. (2004). ORI introduction to the responsible conduct of research. Department of health and human services, office of the secretary, office of Public health and science. Office of Research Integrity.

[CR39] Swift, J. K., Christopherson, C. D., Bird, M. O., Zöld, A., & Goode, J. (2022). Questionable research practices among faculty and students in APA-accredited clinical and counseling psychology doctoral programs. *Training and Education in Professional Psychology*, *16*, 299–305.

[CR40] Ulrich, R., & Miller, J. (2020). Questionable research practices may have little effect on replicability, *eLife 9*. 10.7554/eLife.58237 PMC7561355 32930092

[CR41] van Swol, L. M., Polman, E., Paik, J. E., & Chang, C.-T. (2022). Effects of gain/loss frames on telling lies of omission and commission. *Cognition & Emotion*, *36*, 1287–1298. 10.1080/02699931.2022.2105307 35881056

[CR42] Xie, Y., Wang, K., & Kong, Y. (2021). Prevalence of research misconduct and questionable research practices: A systematic review and meta-analysis. *Science and Engineering Ethics*, *27*. 10.1007/s11948-021-00314-9 34189653

