# Supplementary material for: Questionable Research Practices: A Principled Classification and Ranking Based on Survey Data
Source: Sci Eng Ethics. 2026 Apr 10;32(3):23. doi: 10.1007/s11948-026-00589-w (PMC13230259; doi:10.1007/s11948-026-00589-w)
Supplement: Supplementary file 1 — Supplementary Material 1 [file 11948_2026_589_MOESM1_ESM.pdf]

Supplementary Information:
Exact text used by surveys to define each of the QRPs, and their classificaiton according to the methods used in this study.

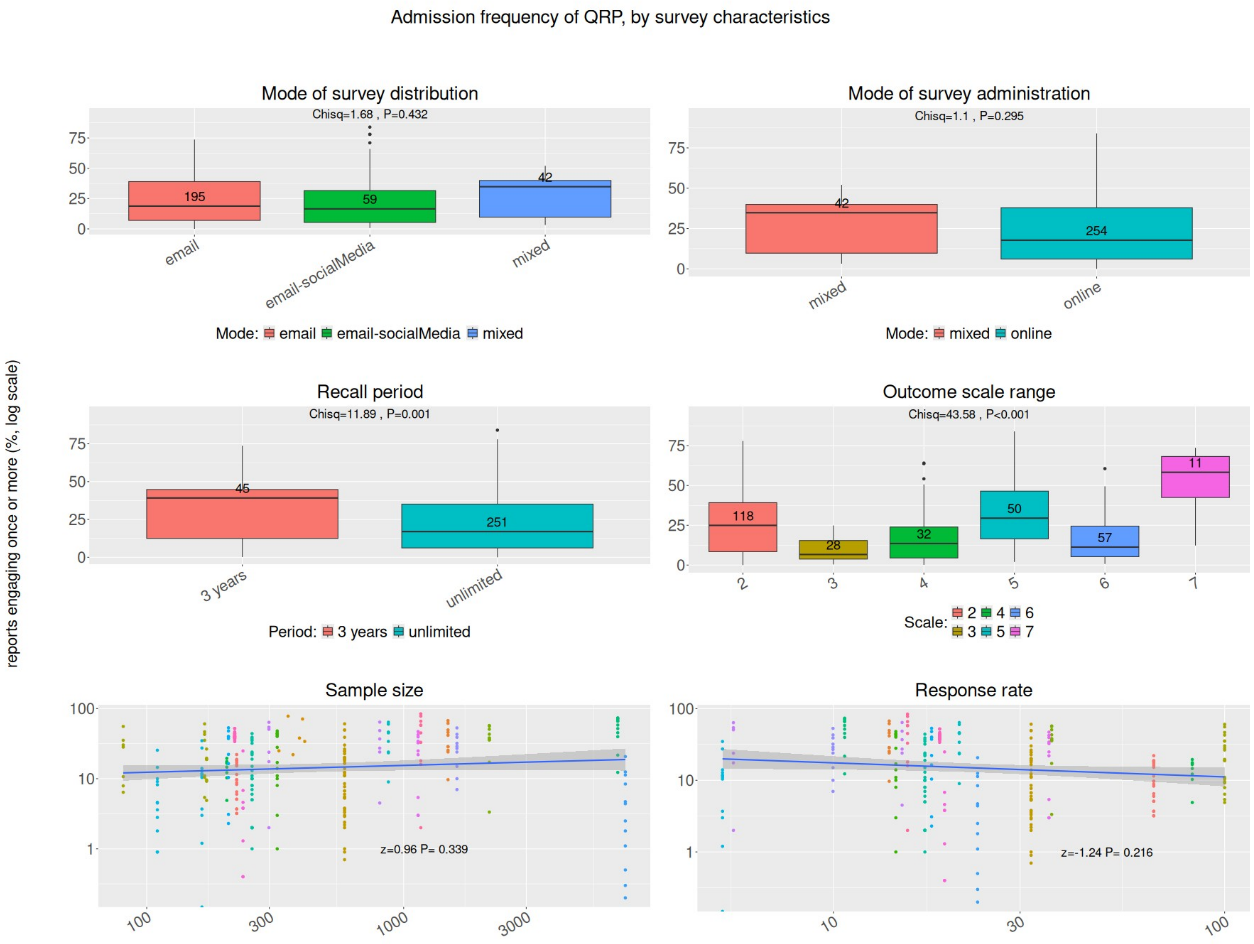

Figure S1: Frequency with which categories of QRP are admitted, by characteristics of survey. Data aggregates results across all QRP categories. Numbers above the median line indicate the number surveys (data points) in the subset. The chi-squared values test for differences between the group medians; the z-tests are from a bivariate linear regression.

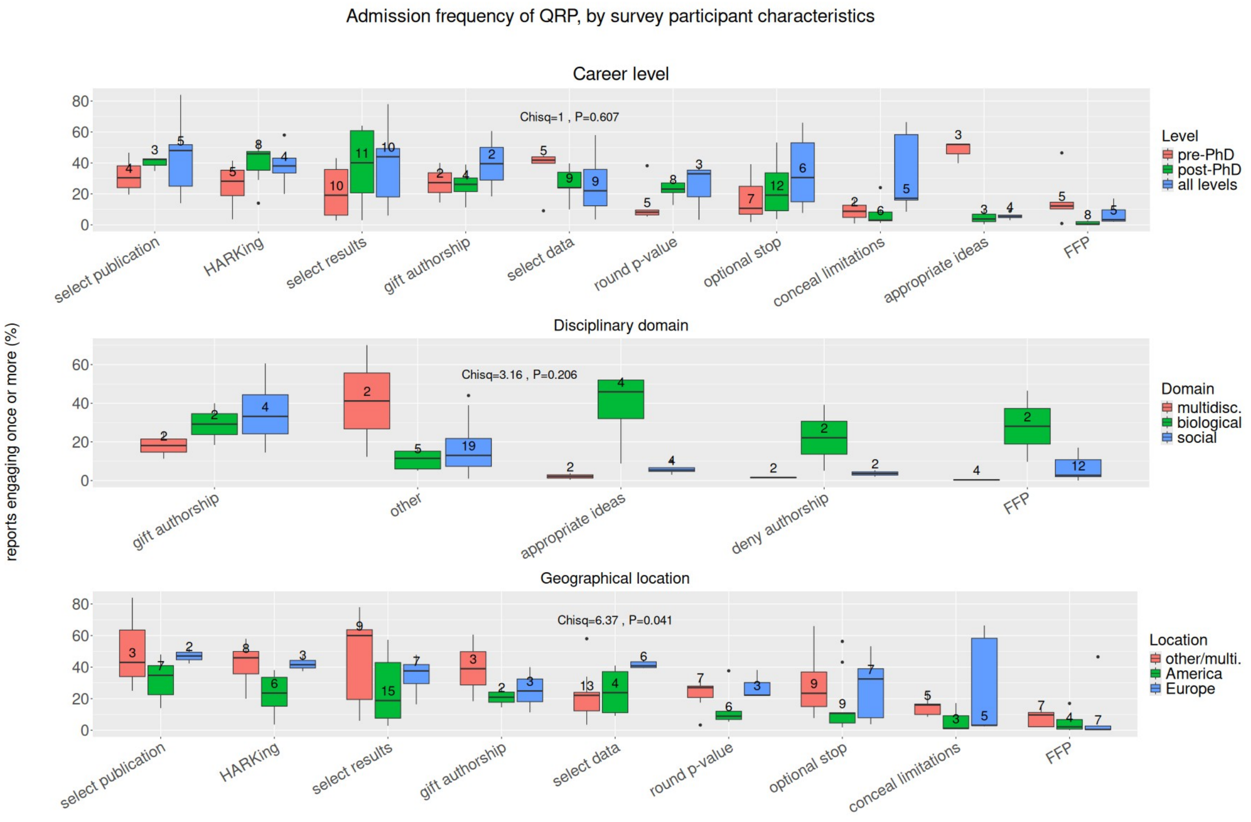

Figure S2: Frequency with which categories of QRP are admitted, by characteristics of survey participants. Data is limited to QRP categories that included at least two data points in each level of the subset. Numbers above the median line indicate the number surveys (data points) in the subset. The chi-squared tests for differences between the group medians.

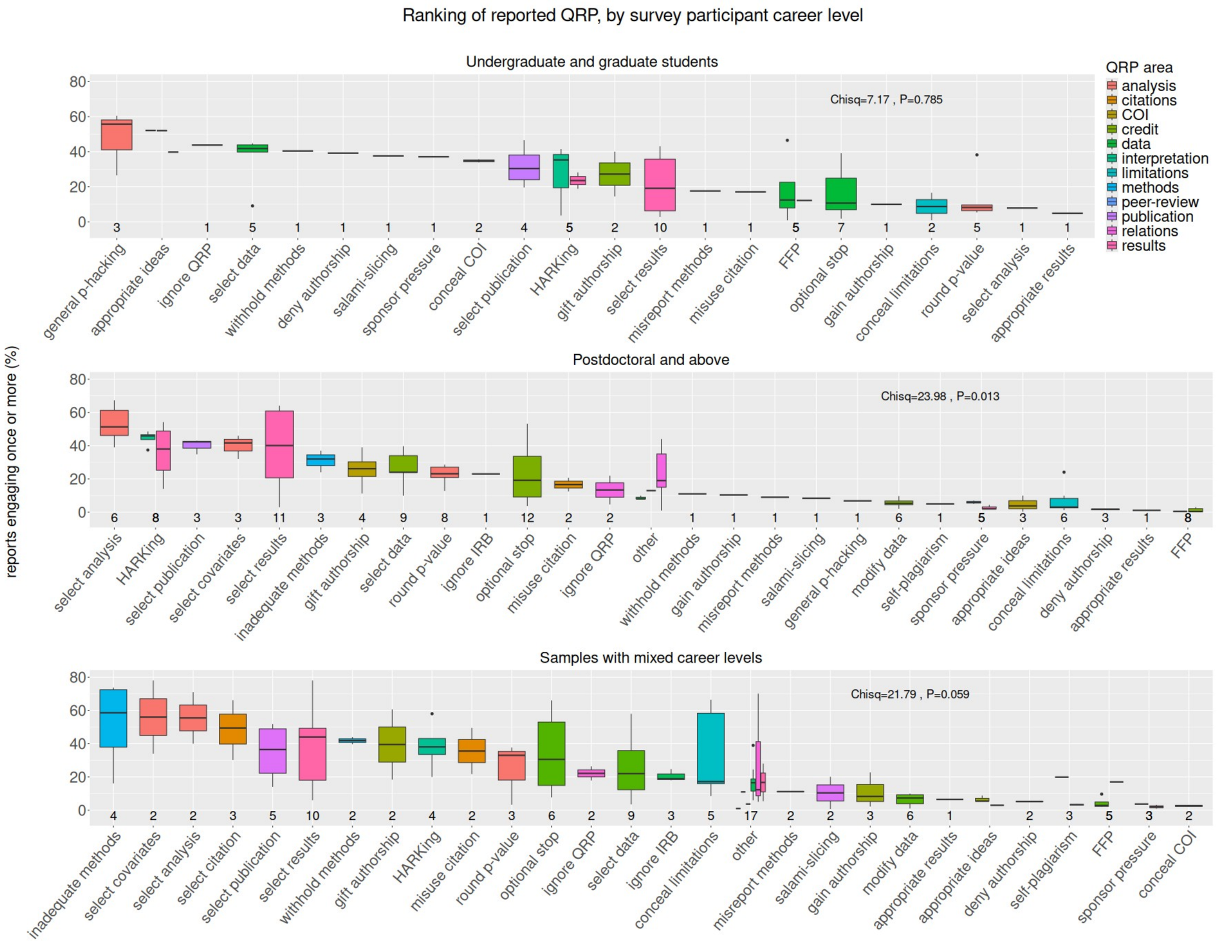

Figure S3: Rank ordering of QRP by median frequency of admission, recalculated for surveys involving participants at different career levels. Numbers in parentheses below each boxplot show the number of data points forming the boxplot. The chi-squared tests for differences between the group medians.

Figure S3: Rank o
involving partici
the number of da
group medians.
